# Systematic Review on the Correlation of Quantitative PCR Cycle Threshold Values of Gastrointestinal Pathogens With Patient Clinical Presentation and Outcomes

**DOI:** 10.3389/fmed.2021.711809

**Published:** 2021-09-23

**Authors:** Stéphane Bonacorsi, Benoit Visseaux, Donia Bouzid, Josep Pareja, Sonia N. Rao, Davide Manissero, Glen Hansen, Jordi Vila

**Affiliations:** ^1^Department of Microbiology, Robert Debré University Hospital, AP-HP, Paris, France; ^2^Université de Paris, IAME, INSERM, Paris, France; ^3^Université de Paris, Laboratoire de Virologie, Hôpital Bichat Claude Bernard, Assistance Publique-Hôpitaux de Paris, Paris, France; ^4^Université de Paris, Service d'Accueil des Urgences, Hôpital Bichat Claude Bernard, Assistance Publique-Hôpitaux de Paris, Paris, France; ^5^STAT-Dx Life, S.L. (a QIAGEN Company), Medical Affairs, Barcelona, Spain; ^6^QIAGEN Inc., Medical Affairs, Germantown, MD, United States; ^7^QIAGEN Manchester Ltd, Medical Affairs, Manchester, United Kingdom; ^8^Microbiology and Molecular Diagnostics, Hennepin County Medical Center, Department of Infectious Diseases, School of Medicine, University of Minnesota, Minneapolis, MN, United States; ^9^Department of Pathology and Laboratory Medicine, School of Medicine, University of Minnesota, Minneapolis, MN, United States; ^10^Biomedical Diagnostic Centre, Department of Clinical Microbiology, Institute of Global Health, School of Medicine, Hospital Clinic, University of Barcelona, Barcelona, Spain

**Keywords:** cycle threshold, pathogen load, gastrointestinal pathogens, systematic review, qPCR, clinical outcomes

## Abstract

**Background:** Quantitative (q) polymerase chain reaction (PCR) cycle threshold (Ct) values represent the number of amplification cycles required for a positive PCR result and are a proxy of pathogen quantity in the tested sample. The clinical utility of Ct values remains unclear for gastrointestinal infections.

**Objectives:** This systematic review assesses the global medical literature for associations between Ct values of gastrointestinal pathogens and patient presentation and clinical outcomes.

**Data Sources:** MEDLINE, EMBASE, Cochrane library databases: searched January 14–17, 2020.

**Study Eligibility Criteria:** Studies reporting on the presence or absence of an association between Ct values and clinical outcomes in adult and pediatric populations were included. Animal studies, reviews, meta-analyses, and non-English language studies were excluded.

**Participants:** Humans infected with gastrointestinal pathogens, detected with qPCR.

**Interventions:** Diagnostics assessing Ct values. Extracted data were reported narratively.

**Results:** Thirty-three eligible studies were identified; the most commonly studied pathogens were *Clostridioides difficile* (*n* = 15), norovirus (*n* = 10), and rotavirus (*n* = 9). Statistically significant associations between low *C. difficile* Ct values and increased symptom severity or poor outcome were reported in 4/8 (50%) studies, and increased risk of death in 1/2 (50%) studies; no significant associations were found between Ct value and duration of symptoms or length of hospital stay. Among studies of norovirus, 5/7 (71%), mainly genogroup II, reported symptomatic cases with significantly lower median Ct values than controls. Significantly lower rotavirus Ct values were also observed in symptomatic cases vs. controls in 3/7 (43%) studies, and associated with more severe symptoms in 2/2 studies. Contradictory associations were identified for non-*C. difficile* bacterial and parasitic pathogens.

**Conclusions:** In conclusion, some studies reported clinically useful associations between Ct values and patient or healthcare outcomes; additional, well-designed, large-scale trials are warranted based on these findings.

**Systematic Review Registration:** [PROSPERO], identifier [CRD42020167239].

## Introduction

Gastrointestinal infections contribute significantly to the burden of illness from infectious diseases worldwide (1, 2). Rotavirus is the principal cause of diarrhea mortality, responsible for a high attributable fraction among all age groups (13.9%) (3). *Shigella*, the second most common cause of diarrhea mortality, is a key contributor to diarrheal death among children younger than 5 years (14.3%), mainly in low income countries (3).

Quantitative (q) polymerase chain reaction (PCR) is a robust and increasingly common methodology for rapid syndromic testing due to its sensitivity and specificity for identification of pathogens. In infectious diseases, qPCR cycle threshold (Ct) values represent the number of amplification cycles required for the fluorescent signal to exceed the basal threshold level. Ct values are inversely related to the number of copies of the target gene in a sample, meaning that lower Ct values correlate with higher pathogen loads. In infectious diseases, qPCR Ct values have potential utility in providing clinicians with information regarding genomic load that may help guide clinical and infection-control decisions. In addition, Ct values may help to clarify diagnostic uncertainty in cases where there is difficulty interpreting binary results, for example when distinguishing between causative infectious pathogen and asymptomatic carriage/colonization (4–6), particularly as identification of multiple pathogens is common (7, 8).

Notably, unprecedented challenges from the COVID-19 pandemic have raised the interest in clinical and diagnostic utility of Ct values (9, 10). However, in a recent systematic review of the utility of Ct values in respiratory infections (parallel to this study), no universal conclusions could be reached [In press: J Antimicrob Chemother 2021]. This systematic review assesses the global medical literature for associations between Ct values of gastrointestinal pathogens and patient or healthcare outcomes.

## Methods

This systematic review was undertaken according to the principles outlined in the Cochrane handbook and guidance published by the Center for Reviews and Dissemination. The original protocol was published in the PROSPERO database (CRD42020167239) and included broad search terms unrestricted by pathogen or disease type. This review focuses on gastrointestinal pathogens.

### Eligibility Criteria

Literature searches of MEDLINE, EMBASE, and the Cochrane Library using search tools at ncbi.nlm.nih.gov/pubmed, embase.com and cochranelibrary.com were undertaken to identify studies reporting on the presence or absence of an association between qPCR Ct values and patient or healthcare outcomes (see Supplementary Table 1 for the PubMed search strategy). The search strategy comprised three concepts: (real-time [rt]-PCR OR qPCR) AND Ct values AND pathogen. Randomized-controlled, single-arm, non-randomized comparative and observational (retrospective or prospective) studies were included. Animal studies, systematic reviews, non-systematic reviews and meta-analyses were excluded; however, additional publications were identified by manual citation searching of appropriate reviews. Searches were limited to English language studies, for reasons of feasibility.

### Study Selection and Data Extraction

Titles and abstracts were screened, based on eligibility criteria, for inclusion by two independent reviewers who then assessed the full texts of relevant studies; a third reviewer resolved conflicts. Key data from all included studies were captured by one reviewer, and subsequently verified by another reviewer. Outcomes were broadly divided into the following categories: mortality, symptomatic vs. asymptomatic, severity of symptoms, duration of symptoms, intensive care unit (ICU) admission, hospitalization and length of stay (LOS).

The quality and risk of bias of each study was assessed using a tool relevant for each study design (Newcastle Ottawa Scale for cross-sectional, cohort, and case-control studies (11).

## Results

### Overview of Studies Included

**Figure 1:**
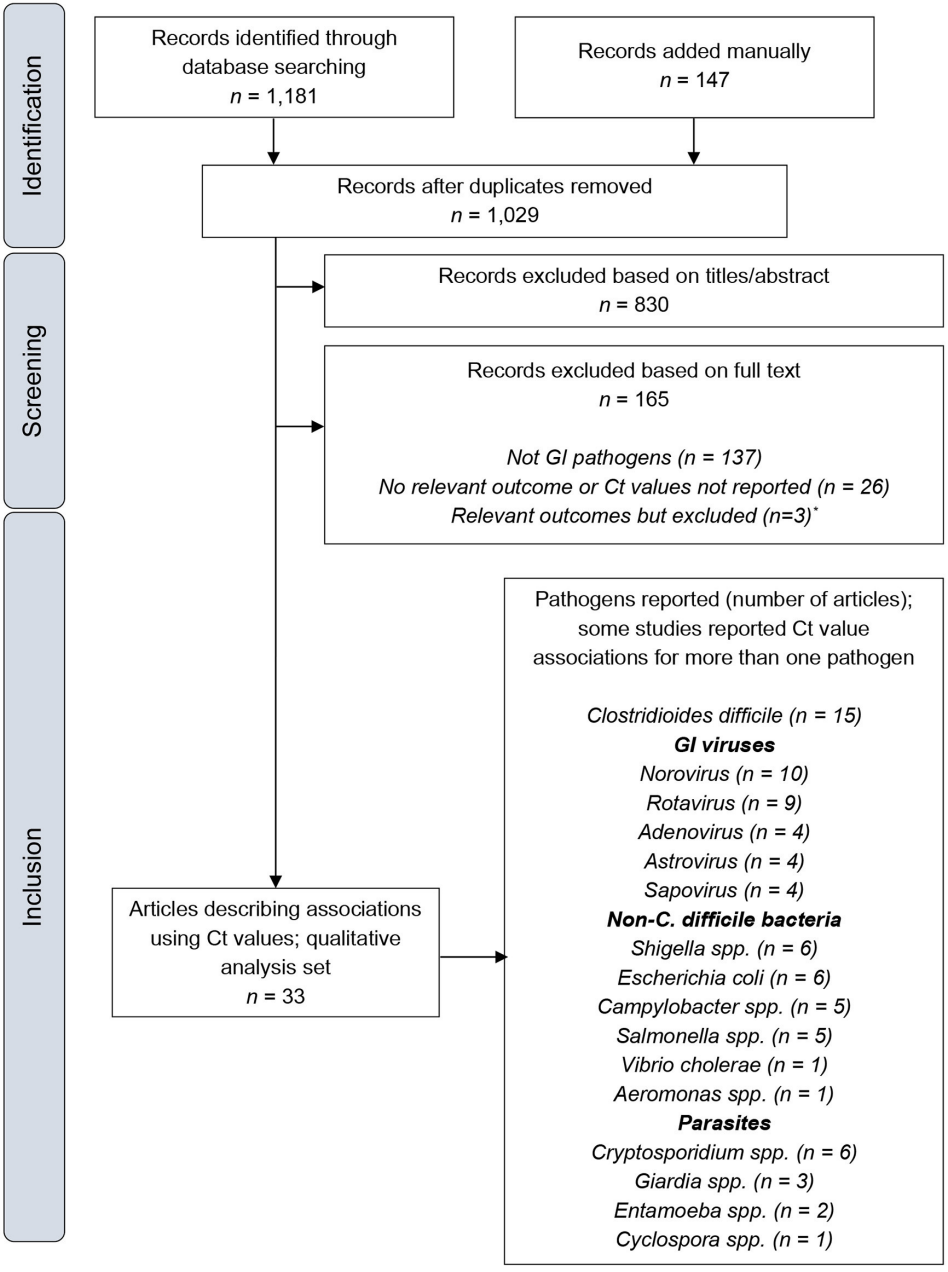
PRISMA flow diagram. Ct, cycle threshold; GI, gastrointestinal. *Details of these publications are provided in the Supplementary Material.

Literature searches, conducted January 14–17, 2020, identified 1,029 unique records. Application of distinct screening and restriction parameters specific to gastrointestinal infections identified 33 eligible studies. Most studies reported Ct value association for more than one pathogen; the most commonly studied pathogens were *Clostridioides difficile* (*n* = 15), norovirus (*n* = 10), and rotavirus (*n* = 9) (Figure 1). All studies identified gastrointestinal pathogens from stool samples. In studies of *C. difficile*, the majority used genes encoding toxin A or B as PCR targets.

The majority of outcomes reported were related to symptoms, including symptom severity, symptomatic vs. asymptomatic and duration of symptoms. Mortality was assessed by three studies. No studies investigated associations between Ct values and hospitalization and/or ICU admission. The majority (84.8%; 28/33) of studies did not report normalized Ct values. Some (66.7%; 22/33) studies presented Ct value distributions.

### Quality and Bias

Using Newcastle-Ottawa scales, all cross-sectional studies, cohort studies and case-control studies were classed as being of poor quality (Supplementary Tables 2–4, respectively). This was generally due to a lack of comparability between groups, insufficient or unjustified sample sizes, the use of non-representative samples (often hospitalized patients or age-specific populations) and a lack of detail regarding patient follow-up or non-response; ascertainment of exposure and outcome was usually appropriate. In an assessment of qPCR methodology, 14/29 (48%) full-length articles were considered to have some or many gaps in the reported methodology (Supplementary Table 5).

### Clostridioides difficile

*C. difficile* was the most commonly reported gastrointestinal pathogen with respect to articles describing associations between Ct values and patient or healthcare outcomes (Table 1). Two studies investigated the association of Ct value with mortality, of which one (*N* = 1,013) reported no significant associations (12). The second study, conducted by Davies et al. at four UK hospitals, was the largest *C. difficile* assessment in this systematic review (*N* = 1,281). The authors reported significantly lower median Ct values for patients who died with *C. difficile* infection compared with those who survived [25.5 (*n* = 123) vs. 27.5 (*n* = 762), respectively; *p* = 0.021] (13).

**Table 1:** Summary of studies that assessed PCR Ct values for *C. difficile* infections against patient clinical presentation and outcomes.

**Outcome**	**Study**	**Number of PCR+ patients**	**Population**	**Outcome measure (significant associations bolded)**
Mortality	Davies et al. Plos One 2018	1,281	UK, hospital	• **Lower median Ct values for patients who died (*****n*** **=** **123) with infection compared with those who survived (*****n*** **=** **762; median Ct 25.5 and 27.5, respectively;** ***p*** **=** **0.021)** • **Following optimal cut-off determination, low Ct was defined as** **≤25 and was significantly associated with mortality (*****p*** **=** **0.032)**
	Rao et al. CID 2015	1,013	USA, hospital, ≥18 years	• There was no association between Ct values and 30-day mortality [OR 1 (95% CI 0.93–1.08); *p* = 0.95]
Severity of symptoms	Rao et al. CID 2015	1,013	USA, hospital, ≥18 years	• There was no association between Ct values and severe CDI [OR 1.01 (95% CI 0.93–1.09); *p* = 0.873]
	De Francesco et al. Anaerobe 2019	421	Italy, hospital and community	• **Ct values** ** <25 were significantly associated with severe disease vs. mild/moderate disease [97 (55%) vs. 79 (44%);** ***p*** **=** **0.0075]** • **Ct values** **>25 were significantly associated with mild/moderate disease vs. severe disease [143 (58.3%) vs. 102 (41.6%);** ***p*** **=** **0.004]** • **The median Ct values of** ***tcdB*** **PCR in patients with mild/moderate disease were significantly higher (28.1; IQR 7.7) than in patients with severe disease (25.9; IQR 5.9) (*****p*** **=** **0.00001)** • **A Ct value** **≤26 was significantly associated with patients with a severe disease**
	Origüen et al. JCM 2019	219	Spain, hospital, ≥18 years	• The mean PCR Ct was lower for patients with a poor outcome (24.9 ± 4.24 vs. 26.05 ± 4.47; *p* = 0.07) (“Poor outcome” was defined as the occurrence of a severe or severe-complicated first CDI episode and/or all-cause death within the first 8 weeks after the end of treatment) • The optimal cut-off Ct value was established as 27.55, yielding a sensitivity of 78.6% (95% CI 67.1–87.5), a specificity of 35.7% (95% CI 28.2–43.7), a PPV of 35.3% (95% CI 31.5–39.2), and an NPV of 78.9% (95% CI 69.5–85.9)
	Kamboj et al. J Infect 2018	183	USA, tertiary care cancer hospital	• **Severe and complicated infections were associated with lower Ct values than non-severe infections [median Ct values for non-severe Ct** **=** **28.0 (*****n*** **=** **168), severe Ct** **=** **24.5 (*****n*** **=** **11), and complicated Ct** **=** **22.5 (*****n*** **=** **4);** ***p*** **=** **0.005]**
	Reigadas et al. J Antimicrob Chemother 2016	Derivation cohort: 129 Validation cohort: 170	Spain, hospital, ≥17 years	Derivation cohort • **Ct value was independently associated with poor outcome by multivariate analysis [OR 0.701 (95%CI 0.604–0.813);** ***p*** **<** **0.001]** • Patients were classified into risk categories; high risk of poor-outcome (Ct <23.5 cycles); medium risk of poor-outcome (Ct 23.5–27.9 cycles); and low risk of poor-outcome (Ct ≥28.0 cycles). The sensitivity of the rule was 46.5% (95% CI 32.5–61.1) and specificity was 98.8% (95% CI 93.7–99.8), the PPV was 95.2% (95% CI 86.1–100) and NPV was 78.7% (95% CI 70.9–86.4); the diagnostic accuracy was 81.4% (95% CI 74.7–88.1) • **Patients with poor-outcome CDI episodes had lower median Ct values than those without poor-outcome CDI episodes (24.8 vs. 28.9;** ***p*** **<** **0.001)**
				Validation cohort
				• **Median Ct value was lower for episodes with poor outcome than favorable (21.9 vs. 27.0;** ***p*** **<** **0.001)** • **Independent association between Ct value and poor outcome (*****p*** **<** **0.001) and the high-risk category (Ct** ** <23.5) and poor outcome (*****p*** **<** **0.001)**
	Anikst et al. Diag Microbiol Infect Dis 2016	118	USA^*^, adults	• No difference in organism burden between groups with (*n* = 59) and without (*n* = 59) clinically significant diarrhea [median Ct, 26.9; (IQR 23.9–32.2) vs. 27.1 (IQR 23.4–30.7); *p* = 0.25; mean Ct 27.9 vs. 27.4]
	Jazmati et al. Clin Microbiol Infect 2016	99	Germany, hospital	• **Patients with severe disease had significantly lower Ct values compared with non-severe infections [26.5** **±** **4.8, (*****n*** **=** **9) vs. 31.2** **±** **4.8 (*****n*** **=** **45);** ***p*** **=** **0.02], describing lower Ct values as a predictor of severe disease (area under the receiver operating characteristic curve 0.77, 95% CI 0.62–0.92;** ***p*** **=** **0.013)**
	Sante et al. Enferm Infecc Microbiol Clin 2018	62	Spain, hospital	• The was no significant difference between Ct values of patients with and those without serious disease [27 ± 4 (*n* = 42) vs. 29 ± 14 (*n* = 20), respectively; *p* = 0.23]
Case vs. control	Crobach et al. J Clin Microbiol 2018	208	Netherlands, hospital	• Comparable mean Ct values were observed for symptomatic patients with subsequent negative toxin A/B immunoassay results [30.4 (95% CI 29.5–31.3)] and asymptomatic carriers [29.2 (95%CI 27.3–31.2)], while **symptomatic patients with positive toxin A/B results had significantly lower mean Ct values, according to ANOVA [24.4 (95% CI 23.5–25.3);** ***p*** **<** **0.001]**
	Hecht et al. Open Forum Infect Dis 2019	193	USA^*^, <3 years	• **Among six (4%) patients who met strict criteria for true infection (including consistent clinical syndrome with no alternative etiology of diarrhea), median Ct value (IQR) was significantly lower than those who did not meet the criteria (classed as colonized) 23.8 (22.0–29.5) vs. 30.5 (26.3–35.8)**, ***p*** **=** **0.03**
	Bruijnesteijn van Coppenraet et al. Clin Microbiol Infect 2015	85	Netherlands, primary Care	• No significant difference in Ct values across all case subjects and controls; however, Ct values were significantly lower (*p* <0.05) in cases vs. controls for patients aged 21–50 (*n* = 19)
	Bub et al. Tropical Medicine and International Health 2017	13	Ivory Coast	• There was a trend toward lower median Ct values in symptomatic patients vs. controls (32 vs. 36, respectively)
Duration of symptoms	Feghaly et al. CID 2013a	120	USA, hospital, adult	• When patients were segregated into quartiles based on their initial Ct values, there was no difference in the time to diarrhea resolution among patients
	Feghaly et al. J Ped 2013b	74	USA, hospital, pediatric	• When patients were segregated into quartiles based on their initial Ct values, there was a paradoxical trend toward a longer interval to diarrhea resolution in children with a lower bacterial burden at diagnosis (*p* = 0.06) • Lower fecal bacterial burden at diagnosis (as calculated by Ct value) was associated with longer times to diarrhea resolution (HR 0.93; 95% CI 0.86–1; *p* = 0.058)
Recurrence	Origüen et al. JCM 2019	219	Spain, hospital, ≥18 years	• **The mean Ct value was lower in patients with recurrence compared with those without (24.00** **±** **3.28 vs. 26.02** **±** **4.54;** ***p*** **=** **0.002)**
Median LOS	Davies et al. Plos One 2018	1,281	UK, hospital	• Patients with low Ct values (≤ 25) had a numerically greater LOS compared with those with high Ct values (>25); however this difference was not significant (Ct ≤ 25: 28 days vs. Ct >25: 23 days; *p* = 0.77) • **In patients with presence of PCR-ribotype 027, LOS was significantly increased in those with low vs. high Ct (32.5 days vs. 28 days;** ***p*** **=** **0.018)**
Other biomarker	Davies et al. Plos One 2018	1,281	UK, hospital	Lower Ct values were associated with: • Higher mean white cell count (Ct ≤ 25: 12.1 ×10^9^/L vs. Ct >25 10.9 ×10^9^/L; *p* = 0.3) • **Higher baseline mean serum creatinine (Ct** **≤25: 120.0 mg/dL vs. Ct** **>25: 110.7 mg/dL;** ***p*** **=** **0.04)** • Lower mean serum albumin (Ct ≤ 25: 31.3 g/L vs. Ct >25: 32.4 g/L; *p* = 0.34)
	De Francesco et al. Anaerobe 2019	421	Italy, hospital and community	• **Statistically significant correlation between low Ct values and leucocytosis (*****p*** **<** **0.001)** but not with the alteration in baseline creatinine or serum albumin level

*Text in bold indicates a statistically significant association*.

*ANOVA, analysis of variance; CDI, C. difficile infection; CI, confidence interval; Ct, cycle threshold; HR, hazard ratio; IQR, interquartile range; LOS, length of stay; NPV, negative predictive value; OR, odds ratio; PCR, polymerase chain reaction; PPV, positive predictive value; UK, United Kingdom; USA, United States of America*.

* *Likely setting although not confirmed in source material*.

Among 12 articles reporting associations between Ct values and symptoms, eight investigated severity of symptoms. Three studies reported significantly lower median Ct values in patients with severe or complicated disease vs. those with mild/moderate disease: De Francesco et al. (*N* = 421) severe 25.9 (*n* = 199) vs. mild/moderate 28.1 (*n* = 222), *p* = 0.00001; Jazmati et al. (*N* = 99) severe 26.5 vs. mild/moderate 31.2, *p* = 0.02; Kamboj et al. (*N* = 183) severe 24.5, complicated 22.5, and non-severe 28.0, *p* = 0.005 (14–16). Jazmati et al. further described lower Ct values as a predictor of severe disease [area under the receiver operating characteristic curve 0.77, 95% confidence interval (CI) 0.62–0.92; *p* = 0.013] (14). Reigadas et al. (*n* = 299) showed that Ct value was independently associated with poor outcome (*p* < 0.001) and classified patients into risk categories accordingly; high risk of poor outcome (median Ct <23.5); medium risk of poor outcome (median Ct 23.5–27.9); and low risk of poor outcome (median Ct ≥28.0) (17). A further three studies with numbers of PCR-positive patients ranging from 62 to 219, reported lower Ct values in patients with poorer outcomes or more severe disease; however, differences did not reach statistical significance (18–20).

Four studies investigated differences in Ct values in case vs. control subjects. In Crobach et al. (*N* = 208) mean quantification cycle (Cq) values were significantly lower (*p* < 0.001) in symptomatic patients who were toxin A/B-positive by enzyme immunoassay (24.4, 95% CI 23.5–25.3) than symptomatic patients who were toxin A/B-negative (30.4, 95% CI 29.5–31.3) and asymptomatic carriers (29.2, 95% CI 27.3–31.2) (5). Similar observations were reported in pediatric patients by Bub et al. (*N* = 13; median Ct 32 in symptomatic cases vs. 36 in controls, no significance reported) and Hecht et al. (*N* = 193; median Ct 23.8 in true infections vs. 30.5 in colonized, *p* = 0.03) (6, 21). In a study (*n* = 85) by Bruijnesteijn van Coppenraet et al., although no significant difference in Ct values were observed between cases and controls across all subjects, Ct values were significantly lower in cases vs. controls for age group 21–50 years (22).

Two studies (*N* ≤ 120) investigated association of Ct value with duration of symptoms; no significant associations were reported in either study (23, 24). One large study (*N* = 1,281) of diarrheal patients in the UK investigated Ct value and LOS; however, no significant associations were reported, except for patients with PCR-ribotype 027, where LOS was significantly increased in those with low vs. high Ct value (32.5 vs. 28 days; *p* = 0.018) (13).

### Gastrointestinal Viruses

Associations between patient or healthcare outcomes and the Ct value of gastrointestinal viruses were investigated in 14 studies, with the most commonly studied viruses being norovirus and rotavirus (*n* = 10 and *n* = 9, respectively) (Table 2). The majority of studies (*n* = 10) investigated the difference in Ct values (or viral load derived from Ct values) between cases and controls (symptomatic and asymptomatic, or patients with or without diarrhea).

**Table 2:** Summary of studies that assessed PCR Ct values for gastrointestinal viruses against patient clinical presentation and outcomes.

**Outcome**	**Study**	**Pathogen(s)**	**Number of PCR+ patients**	**Population**	**Outcome measure**			
**NOROVIRUS**								
Mortality	Gustavsson et al. J Clin Virol 2015	Norovirus	534^*^	Sweden, hospital, >60 years	• Ct values were not associated with fatal outcomes [46 patients deceased; HR 0.97 (95% CI 0.92–1.02) per Ct unit decrease; *p* = 0.17]			
Severity of symptoms	Kabayiza et al. Clin Microbiol and Infec 2014b	Norovirus	98	Rwanda, community and hospital, ≤ 5 years	• No significant difference in Ct values was observed for norovirus GI or GII for clinical markers including vomiting, dehydration or intravenous fluid use			
						Vomiting: Yes/No	Dehydration: Severe/moderate/ mild	IV Fluids: Yes/No
		Norovirus GI	22		OR	1.80 *p* = 0.35	0.33 *p* = 0.77	2.08 *p* = 0.13
					Ct	30.2/31.7 *p* = 0.91	34.0/29.4/31/0 *p* = 0.69	30.2/28.3 *p* = 0.37
		Norovirus GII	76		OR	0.84 *p* = 0.51	0.69 *p* = 0.16	0.66 *p* = 0.09
					Ct	27.7/28.6 *p* = 0.83	30.4/27.2/28.4 *p* = 0.54	27.0/28.5 *p* = 0.54
Case versus control	Liu et al. Lancet 2016	Norovirus GII	5,304^†^	Bangladesh, India, Pakistan, The Gambia, Kenya, Mali and Mozambique, community, <5 years	• Norovirus GII showed associations with diarrhea • Cq values <27.6 were defined as “diarrhea-associated” as the 95% CI or the OR was >1. Ct values <23.4 were defined as “highly diarrhea-associated” as the 95% CI or the OR was >2. The ROC cut-off maximally discriminating case-control status was a Ct value of 28.8 (Youden Index 0.15)			
	Saito et al. CID 2014	Norovirus	607 (140 GI, 460 GII, 7 GI/II) infections from 409 patients	Peru, community, infants	• **Median Ct values were lower in diarrheal compared with non-diarrheal samples [orovirus GI: 28.2 vs. 31.0 (*****p*** **<** **0.066); norovirus GII: 26.4 vs. 30.1 (*****p*** **=** **0.0001), respectively]**			
	Phillips et al. BMC Infect Dis 2009a	Norovirus GII	589	England, community/ primary care	• **The median rt-PCR Ct value was significantly lower in infectious intestinal disease cases vs. control**			
						Median [IQR] Ct value: cases	Median [IQR] Ct value: controls	*p*-value
					**All ages**	**34 (25–37)**	**38 (35–39)**	** <0.0001**
					** <5 years**	**34 (26–37)**	**37 (34–48)**	** <0.0001**
					**≥5 years**	**34 (25–38)**	**38 (36–39)**	** <0.0001**
	Dung et al. J Virol Methods 2012	Norovirus GII	138	Vietnam, hospital, ≤ 60 months	• Results are presented in log of target RNA copy number per mL; however, it is noted that these were converted from Cp values using a standard curve • **The viral load of norovirus GII was significantly higher in samples from children with diarrhea (6.85 log/RNA copies/ml; range: 2.89–9.71) than those without (5.07 log/RNA copies/ml; range: 3.63–9.16)** ***p*** **=** **0.02**			
	Kabue et al. J Clin Virol 2016	Norovirus Norovirus GI Norovirus GII	122 (71 GII only, 18 GI only, 33 GI/GII mixed)	South Africa, clinics (community), <5 years	• There was no difference in median Ct value between symptomatic and asymptomatic patients (28.06 vs. 27.58, respectively; *p* = 0.32) • **Significantly lower median Ct values were observed in symptomatic patients compared with asymptomatic patients (27.02 vs. 34.59, respectively;** ***p*** **=** **0.0009)**			
	Kabayiza et al. Pediatr Infect Dis J 2014a	Norovirus GI and GII	GI, *n* = 27 GII, *n* = 51	Rwanda, community and hospital, ≤ 5 years	• **Significantly lower Ct values in patients vs. controls for norovirus GII (25.79 vs. 29.54;** ***p*** **=** **0.04)**, but not for norovirus GI (29.25 vs. 28.94; *p* = 0.08)			
	Elfving et al. JCM 2014	Norovirus GII	37	Zanzibar, community, 2 months−5 years	• There was no significant difference in median Ct values between patients and controls for infections caused by norovirus GII (25.1 vs. 26.9, respectively; *p* = 0.28)			
					• **By multivariate logistic regression analysis (accounting for age and gender), a cut-off Ct value of 45 was associated with disease (OR 10.1; CI 3.5–29.1;** ***p*** **<** **0.0001)**			
Duration of symptoms	Partridge et al. J Hosp Infect 2012	Norovirus	623^‡^	UK, hospital	• No significant correlation was identified between duration of symptoms from time of sampling and Ct value of the sample (Spearman rank correlation coefficient: −0.077; *p* > 0.2)			
Infectiousness	Partridge et al. J Hosp Infect 2012	Norovirus	110	UK, hospital	• There was no significant difference in initial Ct value between onward transmitters and non-transmitters (24.98 vs. 26.56; *p* = 0.19)			
**ROTAVIRUS**								
Severity of symptoms	Kabayiza et al. Clinc Microbiol and Infec 2014b	Rotavirus	325	Rwanda, community and hospital, ≤ 5 years	• **Lower Ct values for rotavirus were significantly associated with multiple clinical markers (vomiting, more severe dehydration and intravenous fluid therapy) in univariate and multivariate analyses**			
						Vomiting: Yes/No	Dehydration: Severe/moderate/mild	IV Fluids: Yes/No
					Univariate analysis			
					OR	**2.80** ***p*** **<** **0.0001**	**2.49** ***p*** **<** **0.0001**	**3.78** ***p*** **<** **0.0001**
					Ct	**21.2/22.3** ***p*** **=** **0.035**	**20.5/21.5/22.8** ***p*** **=** **0.0085**	**20.8/23.0** ***p*** **=** **0.0005**
						Vomiting: Yes/No	Severe dehydration: Yes/No	IV Fluids: Yes/No
					Age-adjusted multivariate analysis			
					OR (CI)	**1.57 (1.04–2.33)** ***p*** **=** **0.032**	**1.47 (0.94–2.44)** ***p*** **=** **0.09**	**2.18 (1.54–3.11)** ***p*** **<** **0.0001**
	Kang et al. J Med Virol 2004	Rotavirus A	91	India, hospital and community, pediatric	• **There was a significant negative correlation (*****r*** **=** **−0.80;** ***p*** **<** **0.001) between symptom severity and the crossing point (Ct value) on the assay, indicating that children with more severe diarrhea have higher viral loads than children with less severe disease** • Mean crossing points (Ct values) in children with high Vesikari severity scores (10–15) were 11.7 (95% CI 10.5–12.9), low severity scores (3–9) were 26.8 (95% CI 24.6–29.0), and asymptomatic children were 35.7 (95% CI 32.5–39.0) • **Significant associations were observed between crossing points and the maximum number of stools in 24 h (*****p*** **<** **0.001), the maximum number of times vomited in 24 h (*****p*** **=** **0.001), and dehydration (*****p*** **=** **0.02)** • No significant associations were found between crossing points and duration of diarrhea, vomiting, or fever			
Case vs. control	Liu et al. Lancet 2016	Rotavirus	5,304^†^	Bangladesh, India, Pakistan, The Gambia, Kenya, Mali and Mozambique, community, <5 years	• **Rotavirus had strong Ct-dependent associations with diarrhea** • Cq values <35.0 were defined as “diarrhea-associated” as the 95% CI or the OR was >1. Cq values <32.6 were defined as “highly diarrhea-associated” as the 95% CI or the OR was >2. The ROC cut-off maximally discriminating case-control status was a Cq value of 26.9 (Youden Index 0.48)			
	Phillips et al. J Clin Virol 2009b	Rotavirus A	153 cases	England, community/ primary care	• **The median rt-PCR Ct value was significantly lower in infectious intestinal disease cases vs. control, both in all ages and when the analysis was restricted to children aged** ** <5 years**			
						Median [IQR] Ct value: cases	Median [IQR] Ct value: controls	*p*-value
					All age groups	**18 (15–30)**	**37 (33–39)**	** <0.0001**
					<5 years	**17 (15–22)**	**37 (33–40)**	** <0.0001**
	Kabayiza et al. Pediatr Infect Dis J 2014a	Rotavirus	238	Rwanda, community and hospital, ≤ 5 years	• No significant difference in median Ct values between patients vs. controls (21.16 vs. 23.29; *p* = 0.24)			
	Dung et al. J Virol Methods 2012	Rotavirus A	113	Vietnam, hospital, ≤ 60 months	• Results are presented in log of target RNA copy number per mL; however, it is noted that these were converted from Cp values using a standard curve • **The viral load of rotavirus A was significantly higher in samples from children with diarrhea (10.6 log/RNA copies/ml; 5.56–12.49) than from those without (8.33 log/RNA copies/ml; 5.43–10.52) (*****p*** **<** **0.001)**			
	Ramani et al. J Med Virol	Rotavirus	103	India, hospital, neonatal	• The mean Ct value was 26.26 (SD 3.06) for symptomatic neonates and 27.34 (SD 2.73) for asymptomatic neonates • There was no significant difference in viral load between symptomatic and asymptomatic neonates (*p* = 0.087) • Neonates with feed intolerance and abdominal distension had significantly lower Ct values than those with other gastrointestinal symptoms (*p* = 0.02)			
	Elfving et al. JCM 2014	Rotavirus	19	Zanzibar, community, 2 months−5 years	• There was no significant difference in median Ct values between patients and controls for infections caused by rotavirus (24.4 vs. 26.0, respectively; *p* = 0.5) • **By multivariate logistic regression analysis (accounting for age and gender), a cut-off Ct value of 45 was associated with disease (OR 5.8; CI 1.7–20.3;** ***p*** **<** **0.003)**			
	Mukhopadhya et al. J Med Virol 2013	Rotavirus	15: 10 symptomatic and 5 asymptomatic	India, hospital and community, <5 years	• The median Cq at presentation in symptomatic children was 17.21 (IQR 14.36–23.96) compared with 30.98 (IQR 29.38–31.50) in asymptomatic children (*p* = 0.086) • **Once removing an outlier in the asymptomatic group, the difference between the initial shedding between symptomatic and asymptomatic samples was found to be statistically significant (*****p*** **=** **0.007)**			
**ALL OTHER GASTROINTESTINAL VIRUSES**								
Severity of symptoms	Kabayiza et al. Clin Microbiol and Infec 2014b			Rwanda, community and hospital, ≤ 5 years	• No significant associations between Ct values and clinical markers were observed for adenovirus, astrovirus or sapovirus			
						Vomiting (Y/N)	Dehydration (Severe/moderate/mild)	IV fluid (Y/N)
		Adenovirus	216		OR	0.77 *p* = 0.18	**0.59** ***p*** **=** **0.014**	**0.70** ***p*** **=** **0.049**
					Ct	36.2/36.1 *p* = 0.91	36.1/36.5/35.9 *p* = 0.73	32.5/31.5 *p* = 0.77
		Astrovirus	36		OR	0.69 *p* = 0.34	1.13 *p* = 0.85	1.67 *p* = 0.17
					Ct	26.7/24.7 *p* = 0.06	26.0/26.7/24.6 *p* = 0.87	26.7/24.9 *p* = 0.45
		Sapovirus	33		OR	0.68 *p* = 0.33	0.44 *p* = 0.32	0.75 *p* = 0.48
					Ct	29.4/29.2 *p* = 0.54	39.1/28.6/26.4 *p* = 0.13	30.6/26.4 *p* = 0.23
Symptomatic vs. asymptomatic (or case versus control)	Liu et al. Lancet 2016	Adenovirus, Sapovirus, *Astrovirus*	5,30	Bangladesh, India, Pakistan, The Gambia, Kenya, Mali and Mozambique, community, <5 years	• **Adenovirus had strong Ct-dependent associations with diarrhea**. Cq values <35.0 were defined as “diarrhea-associated” as the 95% CI or the OR was >1. Cq values <22.7 were defined as “highly diarrhea-associated” as the 95% CI or the OR was >2. The ROC cut-off maximally discriminating case-control status was a Cq value of 30.2 (Youden Index 0.08) • Astrovirus showed associations with diarrhea. Cq values <25.5 were defined as “diarrhea-associated” and <22.2 were defined as “highly diarrhea-associated” (ROC cut-off 28.1; Youden index 0.18) • Sapovirus was only moderately associated with diarrhea. A Cq values <31.6 were defined as “diarrhea-associated” (ROC cut-off 34.1; Youden index 0.02)			
	Kabayiza et al. Pediatr Infect Dis J 2014a			Rwanda, community and hospital, ≤ 5 years	• No significantly lower Ct values in patients vs. controls for other gastrointestinal viruses			
					Median Ct for patients	Median Ct for controls	*p*-value (PCR+)	
		Adenovirus	284		36.27	35.89	0.57	
		Astrovirus	31		25.79	24.15	0.31	
		Sapovirus	38		25.59	26.42	0.94	
	Elfving et al. JCM 2014			Zanzibar, community, 2 months−5 years	• There was no significant difference in median Ct values between patients and controls for infections caused by gastrointestinal viruses			
					Median Ct for patients	Median Ct for controls	*p*-value	
		Adenovirus (any)	98		38.2	39.3	0.05	
		Adenovirus 40/41	16		36.6	35.0	0.66	
		Astrovirus	5		19.9	31.5	–	
		Sapovirus	21		25.6	28.3	0.50	

*CI, confidence interval; Cp, crossing point; Cq, quantification cycle; Ct, cycle threshold; GI/II, genogroup I/II; HR, hazard ratio; IQR, interquartile range; IV, intravenous; OR, odds ratio; PCR, polymerase chain reaction; ROC, receiver operating characteristic; rt, real-time; SD, standard deviation; UK, United Kingdom*.

* *Total number pathogen-specific positive samples in the study; number of PCR+ve samples used in the Ct analysis not provided*.

† *Total number of matched pairs; individual pathogen PCR+ve n-values were not provided, 2,254 samples were positive for one diarrhea-associated pathogen, and 2,063 samples were positive for ≥2*.

‡ *Total number of PCR+ve patients in this study; number of patients included in the analysis of Ct value vs. symptom duration is unclear*.

*Bold indicates a statistically significant association*.

In general, norovirus, particularly norovirus genogroup II (GII), infections were found to have significantly lower median Ct values in infections vs. controls. Kabue et al. (*N* = 122) reported that lower median Ct values were observed in symptomatic pediatric patients compared with asymptomatic pediatric patients infected with norovirus GII (*n* = 104; 27.0 vs. 34.6; *p* = 0.0009) (25). Similar outcomes were reported in Kabayiza et al. (*n* = 51; 25.8 vs. 29.5; *p* = 0.04), Phillips et al. (*n* = 589; 34 vs. 37; *p* < 0.0001), Saito et al. (*n* = 467; 26.4 vs. 30.1; *p* = 0.0001), and Dung et al. (*n* = 138; 6.85 log copies/ml vs. 5.07 log copies/ml; *p* = 0.02) (4, 26–28). Additionally, Liu et al. reported a pathogen quantity-dependent association with diarrhea in children <5 years old (29). Elfving et al. also reported lower median Ct values in patients vs. controls, but the difference was not significant (25.1 vs. 26.9; *p* = 0.28) (30).

One study investigated Ct values of norovirus GII and fatal outcomes (*n* = 534) and found no association (31). One other study reported no significant associations between Ct values and symptom duration (*n* = 623) or infectiousness (*n* = 110) in patients with infections caused by norovirus (32).

Similar to norovirus, multiple studies showed significantly lower Ct values (or Cq) in cases of symptomatic rotavirus infection vs. controls. Phillips et al. (*N* = 153) reported lower median Ct values in rotavirus intestinal infections vs. controls (18 vs. 37; *p* < 0.0001) (33). Dung et al. (*n* = 113) reported significantly higher median viral loads in children with diarrhea compared with those without (10.6 log copies/ml vs. 8.33 log copies/ml; *p* < 0.001) (26), and one study in children <5 years by Liu et al. reported strong pathogen quantity-dependent associations with diarrhea (29). Supporting these observations, Kabayiza et al. (*n* = 325) reported that lower median Ct values were significantly associated with more severe symptoms, including vomiting, severe dehydration and intravenous fluid therapy, in patients with infections caused by rotavirus (27). Kang et al. (*N* = 91) also reported significant associations between severe diarrhea and low Ct values (reported as “crossing points”) (34). Four further studies also reported lower median Ct values in patients vs. controls/asymptomatic patients, but differences did not reach statistical significance: Elfving et al. (*n* = 19; 24.4 vs. 26.0; *p* = 0.50); Kabayiza et al. (*n* = 238; 21.16 vs. 23.29; *p* = 0.24); Ramani et al. (*n* = 103; 26.26 vs. 27.34; *p* = 0.087) and Mukhopadhya et al. (*n* = 15; 17.21 vs. 30.98; *p* = 0.086) (27, 30, 35, 36). Notably, adjustment of an outlier in the study by Mukhopadhya et al. resulted in the difference reaching statistical significance (*p* = 0.007) (36).

Three studies investigated gastrointestinal viruses other than norovirus and rotavirus. In one study that investigated pathogen quantity and diarrhea in children <5 years old, associations between Ct value and diarrhea were reported for cases of adenovirus and astroviruses (29). No other associations between Ct values and cases vs. controls were identified (27, 30).

### Non-*C. difficile* Bacterial and Parasitic Pathogens

Associations between patient clinical outcomes and the Ct value of non-*C. difficile* bacterial and parasitic pathogens were investigated in nine studies (Table 3).

**Table 3:** Summary of studies that assessed PCR Ct values for non-*C. difficile* bacterial and parasitic pathogens against patient clinical presentation and outcomes.

**Outcome**	**Study**	**Pathogen(s)**	**Number of PCR+ patients**	**Population**	**Outcome measure**			
**BACTERIA**								
Severity of symptoms	Kabayiza et al. Clin Microbiol and Infec 2014b			Rwanda, community and hospital, ≤ 5 years	• **Higher pathogen load (lower Ct value) of ETEC-estA**, ***Shigella*** **spp and** ***Campylobacter*** **spp was significantly associated with multiple clinical markers (vomiting, dehydration and intravenous fluid therapy) by age-adjusted multivariate analysis**			
						Vomiting: Yes/No OR (CI)	Severe dehydration: Yes/No OR (CI)	IV Fluids: Yes/No OR (CI)
		ETEC-estA	167			**1.74 (1.08–2.84)** ***p*** **=** **0.024**	0.97 (0.59–1.60) *p* = 0.89	**1.81 (1.20–2.75)** ***p*** **=** **0.004**
		*Shigella* spp	154			1.10 (0.61–1.99) *p* = 0.75	**3.89 (1.23–15.0)** *p* = 0.02	**2.29 (1.21–4.55)** ***p*** **=** **0.01**
		*Campylobacter* spp.	147			**2.21 (1.09–4.63)** ***p*** **=** **0.03**	1.90 (0.82–4.66) *p* = 0.13	1.64 (0.90–3.02) *p* = 0.11
					• **By univariate analysis, lower Ct values were associated with worse symptoms for** ***Campylobacter*** **spp., ETEC-*****eltB*****, ETEC-*****estA**,* **EPEC** ***bfpA*** **and** ***Shigella***			
						Vomiting: Yes/No	Dehydration: Severe/moderate/mild	IV Fluids: Yes/No
		*Campylobacter* spp.	147		Ct	**30.0/34.4** ***p*** **=** **0.017**	29.5/30.5/34.4 *p* = 0.37	29.9/32.3 *p* = 0.26
					OR	1.11 *p* = 0.62	1.13 *p* = 0.12	**1.49** ***p*** **=** **0.03**
		ETEC-*eltB*	275		Ct	32.7/32.7 *p* = 0.22	**31.7/32.8/33.0** ***p*** **=** **0.063**	36.2/37.0 *p* = 0.056
					OR	1.04 *p* = 0.81	1.13 *p* = 0.41	1.03 *p* = 0.83
		ETEC-*estA*	167		Ct	**24.7/33.3** ***p*** **=** **0.0087**	25.5/24.7/32.1 *p* = 0.28	**25.7/31.6** ***p*** **=** **0.032**
					OR	1.36 *p* = 0.15	**2.03** ***p*** **=** **0.0003**	**1.71** ***p*** **=** **0.003**
		EPEC *bfpA*	125		Ct	30.9/33.7 *p* = 0.28	**25.4/30.9/33.1** ***p*** **=** **0.038**	**28.4/33.6** ***p*** **=** **0.0011**
					OR	1.51 *p* = 0.08	0.79 *p* = 0.92	**1.50** ***p*** **=** **0.04**
		EPEC *eae*	222		Ct	34.3/35.9 *p* = 0.85	33.6/34.9/35.0 *p* = 0.30	34.0/35.4 *p* = 0.11
					OR	0.84 *p* = 0.30	0.76 *p* = 0.30	0.73 *p* = 0.051
		*Salmonella*	58		Ct	41.7/40.4 *p* = 0.27	41.7/41.7/40.2 *p* = 0.79	41.7/41.2 *p* = 0.42
					OR	0.87 *p* = 0.65	0.36 *p* = 0.21	0.69 *p* = 0.22
		*Shigella*	154		Ct	28.9/29.2 *p* = 0.66	**25.6/28.3/30.9** ***p*** **=** **0.012**	**27.9/30.5** ***p*** **=** **0.0083**
					OR	**0.68** ***p*** **=** **0.049**	**0.54** ***p*** **=** **0.0003**	**0.47** ***p*** **<** **0.0001**
	Vu DT et al. J Clin Microbiol 2004	*Shigella*	286	Vietnam, community	• **The trend between increasing number of rt-PCR cycles (Ct values) and decreasing disease severity was highly significant (*****p*** **<** **0.001)** • **The number of PCR cycles required to detect a PCR product was highest for patients** **≥5 years with culture-negative, non-bloody diarrheal specimens (36.6) and was lowest for children (<5 years) with culture-positive, bloody diarrheal specimens (25.3) (*****p*** **<** **0.001)**			
Case s. control	Liu et al. Lancet 2016	*Shigella* spp., EIEC, ETEC, *Campylobacter jejuni* or *C coli*, EPEC, *Vibrio cholerae, Salmonella* spp, EAEC, *Aeromonas* spp	5,304^*^	Bangladesh, India, Pakistan, The Gambia, Kenya, Mali and Mozambique, community, <5 years	• ***Shigella*** **spp. or EIEC, and heat-stable ETEC had strong quantity-dependent associations with diarrhea**. • For *Shigella/*EIEC, Cq values <33.1 were defined as “diarrhea-associated” and <27.9 were defined as “highly diarrhea-associated” (ROC cut-off 26.1; Youden index 0.18) • For heat-stable ETEC, Cq values <26.2 were defined as “diarrhea-associated” and <22.8 were defined as “highly diarrhea-associated” (ROC cut-off 25.4; Youden index 0.25) • *Campylobacter jejuni* or *C coli* and EPEC were only moderately associated with diarrhea • For *Campylobacter* spp., Cq values <19.7 were defined as “diarrhea-associated” and <15.4 were defined as “highly diarrhea-associated” (ROC cut-off 25.8; Youden index 0.08) • For EPEC, Cq values <19.5 were defined as “diarrhea-associated” and <16.0 were defined as “highly diarrhea-associated” (ROC cut-off 19.9; Youden index 0.07) • *Vibrio cholerae* and *Salmonella* spp. showed associations with diarrhea • For *Vibrio cholerae*, Cq values <34.9 were defined as “diarrhea-associated” and <33.8 were defined as “highly diarrhea-associated” (ROC cut-off 29.3; Youden index 0.55) • For *Salmonella* spp, Cq values <32.4 were defined as “diarrhea-associated” and <30.7 were defined as “highly diarrhea-associated” (ROC cut-off 29.7; Youden index 0.29) • EAEC and *Aeromonas* spp were associated with diarrhea only in specific study sites or age strata			
	Bruijnestein et al. Clin Microbiol Infect 2015	*Campylobacter* spp. *Salmonella* spp *E.coli* ETEC Typical EPEC Atypical EPEC STEC EAEC	187 32 487 56 20 227 37 127	Netherlands, primary Care	• **Significantly higher relative loads were observed for** ***Campylobacter*** **spp. (*****p*** **<** **0.005)**, ***Salmonella*** **spp. (*****p*** **<** **0.005), ETEC (*****p*** **<** **0.05) and typical EPEC (*****p*** **<** **0.005)** • **Ct values were significantly higher for STEC cases vs. controls (*****p*** **<** **0.05)** • No significant difference in Ct values between cases and controls were observed for EAEC or atypical EPEC			
		S*higella*/EIEC	14					
	Elfving et al. JCM 2014			Zanzibar, community, 2 months−5 years	• **Median Ct values were significantly lower for patients than for controls for infections caused by ETEC-eltB, ETEC-estA and** ***Shigella*** • **By multivariate logistic regression analysis (accounting for age and gender), a cut-off Ct value of 31 was associated with ETEC-*****estA*** **disease (OR 10.1; CI 3.0–34.1;** ***p*** **<** **0.0001) and a Ct value of 30 was associated with** ***Shigella*** **disease (OR 4.3; CI 2.0–9.4;** ***p*** **<** **0.0001)**			
						Median Ct for patients	Median Ct for controls	*p*-value
		*Campylobacter*	112			31.8	33.3	0.12
		ETEC-*eltB*	148			**31.3**	**34.6**	**0.002**
		ETEC-*estA*	94			**32.6**	**37.3**	**0.0001**
		*Salmonella*	13			42.2	40.6	0.22
		*Shigella*	113			**29.2**	**34.5**	**<** **0.0001**
	Kabayiza et al. Pediatr Infect Dis J 2014a			Rwanda, community and hospital, ≤ 5 years	• **Significantly lower Ct values in patients vs. controls for** ***Campylobacter*****, ETEC-*****estA***, but not for other gastrointestinal bacterial infections			
						Median Ct for patients	Median Ct for controls	*p*-value (PCR+)
		*Campylobacter*	121			**29.75**	**33.02**	**0.007**
		ETEC-*eltB*	213			33.91	34.15	0.90
		ETEC-*estA*	130			**24.75**	**34.37**	**0.04**
		EPEC-*bfpA*	66			33.74	33.00	0.52
		EPEC-*eae*	167			34.84	35.95	0.05
		*Salmonella*	46			41.41	40.70	0.23
		*Shigella*	90			30.35	33.99	0.10
	Barletta et al. CID 2011	EPEC	143	Peru, community, <2 years	• **EPEC load was significantly higher in the diarrheal vs. control group (*****p*** **=** **0.016)** • EPEC bacterial load was similar between mild and moderate cases (144 vs. 95 bacteria/mg; *p* = 0.722) • For a given child, the odds of diarrhea increased by 29% (OR, 1.29; 95% CI 1.08–1.53) for each log10 unit increase in bacterial load in the stool sample			
Duration of symptoms	Barletta et al. CID 2011	EPEC	143	Peru, community, <2 years	• No difference in bacterial load as related to the duration of the episode (444 vs. 184 vs. 146 bacteria/mg for 7, 7–14, and >14 days, respectively)			
**PARASITES**								
Severity of symptoms	Kabayiza et al. Clin Microbiol and Infec 2014b	*Cryptosporidium*	69	Rwanda, community and hospital, ≤ 5 years	• **For** ***Cryptosporidium*****, patients who received IV fluids had significantly lower Ct values than those who did not (*****p*** **=** **0.042)**			
						Vomiting: Yes/No	Dehydration: Severe/moderate/mild	IV Fluids: Yes/No
					Ct	36.2/36.2 *p* = 0.54	34.5/36.0/38.0 *p* = 0.09	**35.5/38.0** ***p*** **=** **0.042**
					OR	0.90 *p* = 0.68	0.51 *p* = 0.31	**1.68** ***p*** **=** **0.044**
Case vs. control	Liu et al. Lancet 2016	*Cryptosporidium* spp., *Cyclospora cayetanensis, Entamoeba histolytica*.	5,304^*^	Bangladesh, India, Pakistan, The Gambia, Kenya, Mali and Mozambique, community, <5 years	• ***Cryptosporidium*** **spp, had strong quantity-dependent associations with diarrhea** • Cq values <29.1 were defined as “diarrhea-associated” and <24.0 were defined as “highly diarrhea-associated” (ROC cut-off 27.5; Youden index 0.17) • *Cyclospora cayetanensis* and *Entamoeba histolytica* showed associations with diarrhea • For *Cyclospora cayetanensis*, Cq values <29.6 were defined as “highly diarrhea-associated” (ROC cut-off 34.0; Youden index 0.40) • For *Entamoeba histolytic*, Cq values <34.8 were defined as “diarrhea-associated” and <32.6 were defined as “highly diarrhea-associated” (ROC cut-off 26.9; Youden index 0.48)			
	Bruijnestein et al. Clin Microbiol Infect 2015	*C.parvum/ hominis* *Giardia lamblia*	56 118	Netherlands, primary Care	• **Significantly higher relative loads were observed for** ***C. parvum/hominis*** **(*****p*** **<** **0.05)** • Higher loads were observed for *G. lamblia*, although statistical significance was not reached; *p* = 0.084			
		*D. fragilis*	832		• **Significantly higher Ct values were observed for** ***D. fragilis*** **cases vs. controls (*****p*** **<** **0.05)**			
	Haque et al. Clin Infect Dis 2009			Bangladesh, hospital		Patients	Controls	*p*-value
		*Cryptosporidium parvum*	20		Mean Ct (95% CI)	41.0 (37.5–44.5)	36.3 (29.4–43.1)	0.127
					Median Ct	43.2	33.7	
		*Cryptosporidium hominis*	61		Mean Ct (95% CI)	33.6 (32.0–35.3)	36.5 (33.7–39.3)	0.098
					Median Ct	34.0	35.1	
		*Entamoeba histolytica D*	83		Mean Ct (95% CI)	35.4 (34.3–36.4)	36.5 (35.3–37.6)	0.18
					Median Ct	35.8	36.9	
		*Giardia lamblia A*	42		Mean Ct (95% CI)	**37.4 (34.8–40.1)**	**31.5 (28.2–34.8)**	**0.017**
					Median Ct	**39.4**	**31.1**	
		*Giardia lamblia B*	333		Mean Ct (95% CI)	**34.9 (33.9–36.0)**	**31.2 (30.4–32.1)**	** <0.001**
					Median Ct	**35.9**	**30.6**	
	Forsell et al. Parasites and Vectors 2016	*Giardia intestinalis*	92	Zanzibar, outpatients	• No difference in Ct values was noted in the qPCR for *Giardia intestinalis* when comparing stool samples from patients with or without diarrhea (mean Ct values 28.2 and 28.5, respectively)			
	Elfving et al. JCM 2014	*Cryptosporidium*	67	Zanzibar, community, 2 months−5 years	• **Median Ct values were significantly lower for patients than for controls for infections caused by** ***Cryptosporidiu*****m (32.1 vs. 36.8, respectively;** ***p*** **=** **0.0009)** • **By multivariate logistic regression analysis (accounting for age and gender), a cut-off Ct value of 35 was associated with disease (OR 8.5; CI 3.5–20.6;** ***p*** **<** **0.0001)**			
	Kabayiza et al. Pediatr Infect Dis J 2014a	*Cryptosporidium*	23	Rwanda, community and hospital, ≤ 5 years	• No difference in Ct values between patients and controls (36.59 vs. 39.85, respectively; *p* = 0.12)			

*CI, confidence interval; Cp, crossing point; Cq, quantification cycle; Ct, cycle threshold; EAEC, enteroaggregative E. coli; EIEC, enteroinvasive E. coli; EPEC, enteropathogenic E. coli; ETEC, enterotoxigenic E. coli; IV, intravenous; OR, odds ratio; PCR, polymerase chain reaction; qPCR, quantitative polymerase chain reaction; ROC, receiver operating characteristic; STEC, Shiga toxin-producing E. coli; UK, United Kingdom*.

* *Total number of matched pairs; individual pathogen PCR+ve n values were not provided, 2,254 samples were positive for one diarrhea-associated pathogen, and 2,063 samples were positive for ≥2*.

*Bold indicates a statistically significant association*.

Among bacterial studies, the majority investigated associations between quantitative PCR-derived bacterial loads and cases vs. controls (symptomatic vs. asymptomatic, or patients with vs. without diarrhea), and most studies found significant associations. Among five studies reporting differences in Ct values between cases vs. controls, significantly lower median Ct values were reported in cases of enterotoxigenic *Escherichia coli* (ETEC), enteropathogenic *E. coli* (EPEC), *Campylobacter* spp., enteroinvasive *E. coli* (EIEC)/*Shigella* spp., and *Salmonella* spp. (22, 27, 29, 30, 37). However, associations were not consistent across studies, including two reports (*n* = 9 and *n* = 46) of no significant difference in cases vs. controls for *Salmonella* spp. (27, 30). In one study, associations were notably weaker for *Campylobacter* spp. and typical EPEC (29). In a study (*n* = 143) of patients with EPEC, a 29% increase in risk of diarrhea was observed for each log_10_ unit increase (calculated by Ct value) in bacterial load (OR 1.29; 95% CI 1.08–1.53) (37).

Two studies also investigated associations between Ct values and bacterial disease severity. In cases of EIEC/*Shigella* spp., lower Ct values were significantly associated with higher vs. lower categories of disease severity (*n* = 286; Ct value 25.3 vs. 36.6), dehydration (*n* = 154; OR 3.89; *p* = 0.02), and requirement for intravenous fluids (*n* = 154; OR 2.29; *p* = 0.01) (27, 38). Lower Ct values for ETEC-*estA* were significantly associated with vomiting (*n* = 167; OR 1.74; *p* = 0.024) and with intravenous fluids (*n* = 167; OR 1.81; *p* = 0.004), and *Campylobacter* spp. with vomiting (*n* = 147; OR 2.21; *p* = 0.03) (27).

One study (*n* = 143) investigated the effect of EPEC bacterial load on the duration of symptoms; however, no significant association was observed (37).

All studies of parasites investigated associations between Ct values (or Cq) and cases vs. controls (symptomatic vs. asymptomatic, or patients with vs. without diarrhea). In studies including *Cryptosporidium* spp., two reported significantly lower Ct values in cases vs. controls, including Elfving et al. (*n* = 67; median Ct 32.1 vs. 36.8; *p* = 0.0009) (22, 30). One further study also reported lower Ct values in cases vs. controls (*n* = 23), but did not reach statistical significance (27). Furthermore, and contrary to expected results, Haque et al. reported higher mean Ct values in *Cryptosporidium parvum* and *Cryptosporidium hominus* cases than controls, although the differences were not significant (*p* = 0.127 and 0.098) (39). In a study in children <5 years (*n* = N/A), strong pathogen quantity-dependent associations with diarrhea were reported in cases of *Cryptosporidium* spp. (29). Among three studies including *Giardia* spp. (*n* = 118, *n* = 375 and *n* = *92*), none reported statistically significant lower Ct values in cases vs. controls (22, 39, 40). Notably, Haque et al. reported that *Giardia lamblia* parasite load, as measured by Ct values, was inversely related to diarrhea, which the authors suggest could be related to the primary role played by the immune system in diarrheal illness that results from these infections (39).

## Discussion

The objective of this systematic review was to assess the global medical literature for any correlation between Ct values and clincal outcomes of patients with gastrointestinal infections. Lower Ct values correspond with greater quantities of detectible target gene and therefore a higher pathogen load, which may correspond with less favorable clinical outcomes. Here we report outcomes from studies identified that report on gastrointestinal pathogens only. This review gathers data from 33 studies, with the largest number of studies for *C. difficile* (*n* = 15). The most common outcomes reported were related to symptoms, including case vs. control, with vs. without diarrhea, and severity of symptoms.

Evidence in this review suggests associations between Ct values and symptomatic *C. difficile* infections. Four out of eight studies reporting the association between lower Ct values and increased disease severity found the association to be significant, including two studies that reported lower Ct values as a predictor of poor outcome (14, 17). Furthermore, 2/4 case vs. control studies reported significantly lower Ct values in symtomatic cases. Most of the *C. difficile* studies reported genes encoding toxin A/B as the target for PCR diagnostics, which when detected by other methods, is generally inferred as marker of disease severity (5).

All studies of norovirus and rotavirus reported lower Ct values in cases vs. controls; the majority for norovirus GII and ~50% for rotavirus reported significant differences. Furthermore, two studies of rotavirus infections reported significant associatons between lower Ct values and severity of symptoms, including vomiting, severe dehydration and administering intravenous fluids (27, 34). Notably, the association of Ct values and symptom severity was more pronounced for norovirus GII than norovirus GI (25, 27, 28). One possible explanation for this is the increased virulence observed with GII infection compared with other norovirus genogroups (41), although more investigation is necessary to draw firm conclusions.

This review found less evidence for the clinical utility of Ct values in non-*C. difficile* bacterial and parasitic infections compared with *C. difficile* and gastrointestinal viruses. Multiple studies reported significant associations between bacterial loads and symptomatic cases, particularly for *Shigella* (29, 30). Two studies reported *Shigella* association with symptom severity (27, 38). Inconsistencies were found in studies of parasitic infections; some studies indicated an association between low Ct values and symptomatic infection in patients with *Cryptosporidium* spp., however, evidence is limited (22, 29, 30). There is insufficient evidence to draw conclusions for other parasitic infections.

Among the studies included in this review, evidence suggests that Ct values may have utility in defining symptomatic causality, particularly in cases of polymicrobial infection. In one study of norovirus-positive samples, coinfection with rotavirus was observed in 3.7 and 7.4% of asymptomatic and diarrheal samples, respectively; probable etiology was determined based on relative Ct values, highlighting their utility for defining causitive organisms in this setting (28). Ct values may also aid causative diagnosis in patients with *C. difficille* infection, where asymptomatic colonization (5, 6), and coinfections have been reported (42). *C. difficile* fecal load is already considered to be of diagnostic utility in distinguishing between infection and colonization (43, 44). However, it is essential to consider Ct values within the context of clinical presentation rather than utilize Ct values as an independent marker of disease.

Despite multiple studies reporting significant associations between high genomic load (low Ct values) and symptomatic infections, particularly for *C. difficile*, norovirus and rotavirus, statistically significant evidence was inconsistent across studies despite similar trends. A possible explanation for this is the diversity of populations investigated across each study (e.g., hospital vs. community setting, pediatric vs. adult populations); adjusting for similar settings may uncover stronger trends toward Ct value and patient outcomes. Further assessments of associations between Ct values and LOS, hospital/ICU admission, for example, could also aid in understanding the utility of Ct values in the diagnosis of gastrointestinal infections.

When interpreting the studies in this systematic review, consideration must be given to the settings and populations in which they were conducted. Studies for some pathogens, such as norovirus, were conducted primarily in pediatric populations and as such their conclusions may not apply to adult populations. All but one of the studies investigating non-*C. difficile* bacterial pathogens and parasites were performed in non-industrialized countries; therefore, the clinical impact of Ct values for these pathogens in industrialized countries remains to be determined. Of the seven studies that detected parasites, five investigated a large list of GI pathogens and multiple pathogens were detected for 8–72% patients (22, 27, 29, 30). These studies highlight the utility of syndromic testing in gastrointestinal infection, where multiplex testing is able to detect more pathogens and co-infections than conventional methods (42). It should also be noted that multiplex PCR for GI pathogens does not currently provide a picture of the microbiome, whereas culture-based techniques are able to provide an understanding of dysbiosis resulting from GI infections.

Differences in study methodology and qPCR workflow are likely to impact Ct values, including: specimen source, collection method, transport media type and volume, stability, quality of the sample, time of sampling vs. onset of infection, master mix components, type and concentration of passive reference dye, reaction efficiency, inter- and intravariability in assay platforms, and whether they were single or multiplex systems. Methodologies varied widely between studies and many (39%) had some or many gaps in reporting defined standardized methodologies. Therefore, within-study variability may have limited the ability to detect associations. The majority (84.8%; 28/33) of studies did not report normalized Ct values, which would have provided more accurate estimations of genomic load for each sample. Although outside the scope of our review, we noted not all (66.7%; 22/33) studies presented Ct value distributions. Further studies to understand the distribution of Ct values in relation to patient outcomes across the populations would be necessary if Ct values are to be utilized in clinical decision-making. After data analysis had been completed, we became aware of the Minimum Information for Publication of Quantitative rt-PCR Experiments (MIQE) guidelines (45), which should be applied to laboratory-developed tests. Some of the studies utilized in this review use commercially available assays and, therefore, when implemented in clinical diagnostic routines, applicable validation, and verification using external controls are necessary. Due to the late discovery it was not possible to re-assess the studies using laboratory-developed assays with the MIQE guidelines in mind; however, we believe that assessment of study methodology using these guidelines would not significantly alter the findings of this systematic review.

There were a number of limitations to this systematic review. The protocol restricted articles referring to Ct values as a measure of genomic load, therefore studies which reported genomic load in measures other than Ct value were not picked up in the database searches or excluded from during screening. Furthermore, articles describing Ct values but with no mention of Ct values in the title, abstract or keywords, were not retrieved based on the search parameters used in the database searches. In addition, late in the review we became aware of alternative wording for Ct values, including Cq and “crossing point” [discussed in detail in (45)]; while we have added articles with these terms manually, it is possible that some may have been missed. Another limitation to this review was the assessment of all included studies as poor quality for bias by the Newcastle-Ottawa scale. This is due to the majority of studies reporting Ct values as secondary outputs, as opposed to seeking to compare clinical outcomes against Ct values. Consequently, the studies did not fully align with the risk and bias assessment. There was considerable variability between studies. Given the high heterogeneity between studies, it was not possible to conduct aggregated/meta-analyses, a key limitation in the scope of this review. A number of studies only made comparative analysis between symptomatic and asymptomatic cases, which limits the clinical utility of these studies in defining Ct values as a measure of disease severity. However, Ct values of asymptomatic patients still hold clinical value in order to discriminate between infection and colonization, an observation reported in multiple studies (4–6, 25, 27). A single reviewer conducted the data extraction and a second reviewer checked all the data points. Whilst an acceptable approach, the methodology could have been optimized by double independent reviewer data extraction with a third reviewer for discrepancy resolution. Due to the large number of studies identified as potential data sources for this review, the single-reviewer extraction method ensured that the review remained feasible. Despite these limitations, we believe this review provides insights into the potential clinical utility of gastrointestinal pathogen Ct values. In summary, there is evidence to support relationships between Ct values and clinical outcomes in gastrointestinal infections. Considered alongside clinical presentation, Ct values could help to guide treatment decisions, particularly in cases of *C. difficile*, where treatment is guided by severity of disease and asymptomatic colonization has been observed (5, 6, 46). This review did not uncover sufficient evidence to draw conclusions on the clinical utility of Ct values for non-*C. difficile* bacterial and parasitic infections. This systematic review is the first to assess the relationship between Ct values and clinical outcomes in gastrointestinal infections, large-scale clinical trials with endpoints centered on Ct values are warranted to draw definitive evidence.

## Data Availability Statement

The original contributions presented in the study are included in the article/Supplementary Material, further inquiries can be directed to the corresponding author/s.

## Author Contributions

BV, DB, JP, SR, DM, GH, and JV were involved in conception and design of the study. All authors contributed to interpretation of the data, manuscript drafting and revision, and approved the submitted version.

## Funding

Medical writing support for the development of this manuscript, under the direction of the authors, was provided by Isabella Talbot, BSc, of Ashfield MedComms, an Ashfield Health company, and funded by Qiagen Manchester Ltd. This study received funding from QIAGEN Manchester Ltd. The funder had the following involvement with the study: article processing fees and provision of medical writing support.

## Conflict of Interest

JP, SR, and DM are employed by Qiagen. BV reports grants, personal fees and non-financial support from Qiagen, personal fees and non-financial support from BioMérieux, personal fees from Hologic, personal fees from Gilead, outside the submitted work. DB reports personal fees from Qiagen, outside the submitted work. The remaining authors declare that the research was conducted in the absence of any commercial or financial relationships that could be construed as a potential conflict of interest. The authors declare that this study received funding from Qiagen Manchester Ltd. The funder had the following involvement in the study: article processing fees and provision of medical writing support. The funder was not involved in the study design, collection, analysis, interpretation of data, the writing of this article or the decision to submit it for publication.

## Publisher's Note

All claims expressed in this article are solely those of the authors and do not necessarily represent those of their affiliated organizations, or those of the publisher, the editors and the reviewers. Any product that may be evaluated in this article, or claim that may be made by its manufacturer, is not guaranteed or endorsed by the publisher.
