# Supplementary material for: Systematic Review on the Correlation of Quantitative PCR Cycle Threshold Values of Gastrointestinal Pathogens With Patient Clinical Presentation and Outcomes
Source: Front Med (Lausanne). 2021 Sep 23;8:711809. doi: 10.3389/fmed.2021.711809 (PMC8496934; doi:10.3389/fmed.2021.711809)
Supplement: Supplementary file 1 [file Data_Sheet_1.docx]

**Systematic Review on the Correlation of Real-time PCR Cycle Threshold Values of Gastrointestinal Pathogens with Patient Clinical Presentation and Outcomes**

**Stéphane Bonacorsi, Benoit Visseaux, Donia Bouzid, Josep Pareja, Sonia N. Rao, Davide Manissero, Glen Hansen, Jordi Vila**

***Supplementary Material***

Articles which met many but not all of the inclusion criteria are listed below**.**

Although the following paper made reference to Ct values and gastrointestinal infections, no relevant outcomes to this review were reported:

Phillips, G., Tam, C.C., Conti S., Rodrigues, L.C., Brown, D., Iturriza-Gomara, M., et al. (2010). Community incidence of norovirus-associated infectious intestinal disease in England: improved estimates using viral load for norovirus diagnosis. *Am. J. Epidemiol.* 171, 1014–1022.

The following paper was excluded as we believe the larger study reported in Liu et al. 2016 supersedes this publication:

Lindsay, B., Ochieng J.B., Ikumapayi U.N., Toure, A., Ahmed, D., Li, S., et al. (2013). Quantitative PCR for detection of Shigella improves ascertainment of Shigella burden in children with moderate-to-severe diarrhea in low-income countries, *J. Clin. Microbiol.* 51, 1740-1746.

The following article was excluded as it reports on a smaller follow-up study of GEMS vs. that reported in Liu et al. 2016:

Liu, J., Kabir, F., Manneh, J., Lertsethtakarn, P., Begum, S., Gratz, J., et al. (2014). Development and assessment of molecular diagnostic tests for 15 enteropathogens causing childhood diarrhoea: a multicentre study. *Lancet Infect. Dis.* 14, 716-724.

**Supplementary Table S1.** Search strategy.

| Search | Query |
| --- | --- |
| #1 | Search "Real-Time Polymerase Chain Reaction"[Mesh] OR "Real Time Polymerase Chain Reaction"[all] OR "Real-Time PCR"[all] OR "Real Time PCR"[all] OR "Real-Time PCRs"[all] OR "Kinetic Polymerase Chain Reaction"[all] OR "RT-PCR"[all] |
| #2 | Search "Quantitative Real-Time Polymerase Chain Reaction"[all] OR "Quantitative Real Time Polymerase Chain Reaction"[all] OR "Quantitative Real-Time PCR"[all] OR "Quantitative Real Time PCR"[all] OR "Quantitative Real-Time PCRs"[all] OR qPCR[all] |
| #3 | Search "Cycle Threshold Value"[all] OR "Ct-Value"[all] OR "Ct Value"[all] OR "Cycle Threshold"[all] |
| #4 | Search "Bacterial Load"[Mesh] OR "Viral Load"[Mesh] OR "Infection"[Mesh] OR "Pathogen"[all] OR "Pathogens"[all] OR "Infectious Disease"[all] OR "Viral Load"[all] OR "Bacterial Load"[all] |
| #5 | Search (#1 OR #2) AND #3 |
| #6 | Search #4 AND #5 |
| #7 | Search #4 AND #5 Filters: Review |
| #8 | Search #6 NOT #7 |

**Supplementary Table S2.** Cross-sectional studies assessed by modified Newcastle Ottawa Scale.

| Study | Selection | | | | Comparability | Outcome | | |
| --- | --- | --- | --- | --- | --- | --- | --- | --- |
|  | **1. Representativeness of the sample**  **a) Truly representative of the average in the community***  **b) Somewhat representative of the average in the community***  **c) Selected group**  **d) No description of the derivation of the cohort** | **2. Sample size**  **a) Justified and satisfactory***  **b) Not justified** | **3. Ascertainment of exposure**  **a) Validated measurement tool***  **b) Non-validated measurement tool, but the tool is available or described***  **c) No description of the measurement tool** | **4. Non respondents**  **a) Comparability between respondents and non-respondents characteristics is established, and the response rate is satisfactory***  **b) The response rate is unsatisfactory, or comparability between respondents and non-respondents is unsatisfactory**  **c) No description of the response rate or**  **characteristics of the responders and the non-responders** | **1. Comparability of cohorts on the basis of the design or analysis**  **a) Study controls for the most important factor (select one)***  **b) Study controls for any additional factor***  **c) Cohorts are not comparable on the basis of the design or analysis controlled for confounders** | **1. Ascertainment of outcome**  **a) Independent blind assessment***  **b) Record linkage***  **c) Self-report**  **d) No description** | **2. Statistical analysis**  **a) The statistical test used to analyse the data is clearly described and appropriate, and the measurement of the association is presented***  **b) The statistical test is not appropriate** | **Assessment of quality^†^** |
| Davies et al. Plos One 2018 | - | - | * | N/A | - | - | * | Poor (3*) |
| Crobach et al. J Clin Microbiol 2018 | * | - | * | N/A | - | - | * | Poor (3*) |
| Kamboj et al. J infect 2018 | - | - | * | N/A | - | * | * | Poor (3*) |
| Jazmati et al. Clin Microbiol Infect 2016 | - | - | * | N/A | - | - | unclear | Poor (1*) |
| Rao et al. CID 2015 | - | - | * | N/A | - | - | * | Poor (2*) |
| De Francesco et al. Anaerobe 2019 | * | - | * | N/A | - | - | * | Poor (3*) |
| Sante et al. Enferm Infecc Microbiol Clin 2018 | - | - | * | N/A | - | - | unclear | Poor (1*) |
| Kabue et al. J Clin Virol 2016 | * | - | * | N/A | - | - | * | Poor (2*) |
| Kabayiza et al. Clin Microbiol & Infec 2014b | - | - | * | N/A | - | - | * | Poor (2*) |
| Forsell et al. Parasites & Vectors 2016 | - | - | * | N/A | - | - | * | Poor (2*) |
| Barletta et al. CID 2011 | - | - | * | N/A | - | - | * | Poor (2*) |
| Vu DT et al. J Clin Microbiol 2004 | * | - | * | N/A | - | - | * | Poor (3*) |

*^†^Good: 3 or 4 stars (*) in selection domain AND 1 or 2 stars in comparability domain AND 2 or 3 stars in outcome domain; Fair: 2 stars in selection domain AND 1 or 2 stars in comparability domain AND 2 or 3 stars in outcome domain; Poor: 0 or 1 star in selection domain OR 0 stars in comparability domain OR 0 or 1 stars in outcome domain.*

**Supplementary Table S3.** Cohort studies assessed by Newcastle Ottawa Scale.

| Study | Selection (max. of 5 stars across all of scoring) | | | |  | Comparability (max. of 2 stars) | Outcome (max. of 3 stars across all of scoring) | | | Assessment of quality^†^ |
| --- | --- | --- | --- | --- | --- | --- | --- | --- | --- | --- |
|  | 1) Representative of the exposed cohort: a) Truly representative* b) Somewhat representative of the average in the community* c) Selected group of users d) No description of the derivation of the cohort | 2) Selection of the non-exposed cohort a) Drawn from the same community as the exposed cohort* b) Drawn from a different source c) No description of the derivation of the non-exposed cohort | 3) Ascertainment of exposure a) Secure record* b) Structured interview* c) Written self-report d) No description | 4) Demonstration that outcome of interest was not present at the start of study a) Yes* b) No |  | 1) Comparability of cohorts on the basis of the design or analysis a) Study controls for the most important factor* b) Study controls for any additional factor*  c) Cohorts are not comparable on the basis of the design or analysis controlled for confounders | 1) Assessment of the outcome: a) Independent blind assessment* b) Record linkage* c) Self report d) No description | 2) Was follow-up long enough for outcomes to occur? a) Yes*  b) No | 3) Adequacy of follow-up of cohorts a) Complete follow-up* b) Subjects lost to follow-up unlikely to introduce bias*  c) Follow-up rate <80% and no description  d) No statement |  |
| Reigadas et al. J Antimicrob Chemother 2016 | * | N/A | N/A | N/A |  | - | - | * | * | Poor (3*) |
| Origüen et al. JCM 2019 | - | N/A | N/A | N/A |  | - | - | * | * | Poor (2*) |
| Feghaly et al. CID 2013a | - | N/A | N/A | N/A |  | - | - | * | * | Poor (2*) |
| Feghaly et al. J Ped 2013b | - | - | N/A | N/A |  | - | - | * | N/A | Poor (1*) |
| Gustavsson et al. J Clin Virol 2015 | * | N/A | N/A | N/A |  | - | - | N/A | N/A | Poor (1*) |
| Saito et al. CID 2014 | * | N/A | N/A | N/A |  | - | - | * | - | Poor (2*) |
| Mukhopadhya et al. J Med Virol 2013 | - | * | N/A | N/A |  | - | - | * | - | Poor (2*) |
| Partridge et al. J Hosp Infect 2012 | - | N/A | N/A | N/A |  | - | - | * | - | Poor (1*) |

*^†^Good: 3 or 4 stars (*) in selection domain AND 1 or 2 stars in comparability domain AND 2 or 3 stars in outcome/exposure domain; Fair: 2 stars in selection domain AND 1 or 2 stars in comparability domain AND 2 or 3 stars in outcome/exposure domain; Poor: 0 or 1 star in selection domain OR 0 stars in comparability domain OR 0 or 1 stars in outcome/exposure domain.*

**Supplementary Table S4.** Case-control studies assessed by Newcastle Ottawa Scale.

| Study | Selection (max. of 5 stars across all of scoring) | | | |  | Comparability (max. of 2 stars) | Exposure (max. of 3 stars across all of scoring) | | | Assessment of quality^†^ |
| --- | --- | --- | --- | --- | --- | --- | --- | --- | --- | --- |
|  | 1. Is the case definition adequate?  a) Yes, with independent validation*  b) Yes, e.g. record linkage or based on self-reports  c) No description | 2. Representativeness of the cases  a) Consecutive or obviously representative series of cases*  b) Potential for selection biases or not stated | 3. Selection of Controls  a) Community controls*  b) Hospital controls  c) No description | 4. Definition of Controls  a) No history of disease (endpoint)*  b) No description of source |  | 1. Comparability of cohorts on the basis of the design or analysis a) Study controls for the most important factor* b) Study controls for any additional factor*  c) Cohorts are not comparable on the basis of the design or analysis controlled for confounders | 1. Ascertainment of exposure  a) secure record*  b) structured interview where blind to case/control status*  c) Interview not blinded to case/control status  d) Written self-report or medical record only  e) No description | 2. Same method of ascertainment for cases and controls  a) Yes*  b) No | 3. Non-response rate  a) Same rate for both groups*  b) Non respondents described  c) Rate different and no designation |  |
| Bub et al. Trop Med Int Health 2017 | - | - | * | * |  | - | N/A | N/A | N/A | Poor (2*) |
| Anikst et al. Diag Microbiol Infect Dis 2016 | - | * | N/A | N/A |  | - | N/A | N/A | N/A | Poor (1*) |
| Bruijnesteijn van Coppenraet et al. Clin Microbiol Infect 2015 | - | * | * | * |  | - | N/A | N/A | N/A | Poor (3*) |
| Feghaly et al. J Ped 2013b | - | * | - | N/A |  | - | N/A | N/A | N/A | Poor (1*) |
| Elfving et al. JCM 2014 | - | - | * | * |  | - | N/A | N/A | N/A | Poor (2*) |
| Kabayiza et al. Pediatr Infect Dis J 2014a | - | - | * | * |  | - | N/A | N/A | N/A | Poor (2*) |
| Phillips et al. J Clin Virol 2009b | - | * | * and - | * |  | - | N/A | N/A | N/A | Poor (2/3*) |
| Phillips et al. BMC Infect Dis 2009a | - | * | * and - | * |  | - | N/A | N/A | N/A | Poor (2/3*) |
| Dung et al. J Virol Methods 2012 | - | - | - | * |  | - | N/A | N/A | N/A | Poor (1*) |
| Kang et al. J Med Virol 2004 | - | - | - | - |  | - | N/A | N/A | N/A | Poor (0*) |
| Ramani et al. J Med Virol | - | - | - | - |  | - | N/A | N/A | N/A | Poor (0*) |
| Liu et al. Lancet 2016 | - | * | * | * |  | - | N/A | N/A | N/A | Poor (3*) |
| Haque et al. Clin Infect Dis 2009 | - | * | * | * |  | - | N/A | N/A | N/A | Poor (3*) |

*^†^Good: 3 or 4 stars (*) in selection domain AND 1 or 2 stars in comparability domain AND 2 or 3 stars in outcome/exposure domain; Fair: 2 stars in selection domain AND 1 or 2 stars in comparability domain AND 2 or 3 stars in outcome/exposure domain; Poor: 0 or 1 star in selection domain OR 0 stars in comparability domain OR 0 or 1 stars in outcome/exposure domain.*

**Supplementary Table S5.** Assessment of reported PCR methodology.

| Study | Location | Setting H, hospital; C, community | Solely pediatric patients | When samples were taken | How samples were taken | How samples were stored | Reagents used with samples | Nucleic acid extraction | PCR assay | Who undertook PCR assays | Gaps in methodology: No gaps; minimal gaps; some gaps; many gaps |
| --- | --- | --- | --- | --- | --- | --- | --- | --- | --- | --- | --- |
| Anikst et al. 2016 | USA | H | No | In hospital admission | Leftover stool samples were used | Samples were held in a refrigerator up to 24 hours after routine testing and then aliquots were measured using a digital scale and stored at −80̊C prior to testing | 0.3 grams of the frozen stool sample was emulsified in 1.0mL of sample reagent and centrifuged at 240 × g to remove particulate matter | NR | PCR using the GeneXpert  *C.difficle* Epi assay (Cepheid, Sunnyvale, CA, USA) | Clinical Microbiology laboratory | Minimal gaps |
| Barletta et al. 2011 | Peru | C | Yes | Samples were selected from a passive surveillance diarrhea cohort study | Samples were collected from patients with diarrhea, and randomly  selected healthy children without diarrhea 1 week before  or after the stool sample collection | Fresh stool samples were collected in capped containers and were stored at −20°C until evaluated by PCR | For lysate preparation, 50–300 mg of each stool specimen was dispersed in 250 μL of lysis buffer (0.25% sodium dodecyl sulfate in 0.1 M ethylenediaminetetraacetic acid [EDTA]; pH, 8.0), and 100 μg/mL of proteinase K was added | DNA was isolated from stool samples by cetyltrimethylammonium bromide extraction | qRT-PCR using a iCycler IQTM Multicolor Real-Time PCR Detection System (Bio-Rad, California) monitoring by an  Optical System Software Version 3.1 | NR | Minimal gaps |
| Bruijnesteijn van Coppenraet et al. 2015 | Netherlands | H | No | At GP visit | Stool samples were processed by four laboratories | Aliquoted stool samples were stored at −80°C | NR | For nucleic acid extraction, the sample was suspended in 400 μL STAR buffer (Roche), vigorously shaken on a Magnalyser (1 minute; Roche) and pelleted (3 minutes, 13,000 rpm). A total of 100 μL of supernatant was extracted on the MagnaPure96 (MP96; Roche) using the DNA and Viral NA small volume kit | Multiplex real-time PCR using ABI7500 real-time thermocycler (Life Technologies) | Blinded fashion | Minimal gaps |
| Bub et al. 2017 | Ivory coast | NR | NR | Enrolment | NR | Stool samples were fixed in ethanol and stored in a freezer at –20°C | NR | NR | Multiplex real-time  PCR | NR | NA, conference abstract |
| Crobach et al. 2018 | Netherlands | H | No | Retrospective | NR | Samples were stored at 4°C until testing | Feces samples approximately the size of a pinhead was suspended in 1 ml of stool transport and recovery (STAR) buffer  (Roche Diagnostics, Almere, The Netherlands) and frozen before further processing. After thawing, samples were homogenized in a MagNA Lyser system (Roche Diagnostics, Almere, The Netherlands) (6,000 μ*g* for 30 s) and then centrifuged | DNA extraction was performed using the Nuclisens EasyMag system (bioMérieux, Marcy l’Etoile, France) | Amplification of the *tcdA* and *tcdB* genes was performed with an ABI TaqMan 7,500 real-time PCR system (Applied Biosystems, Nieuwerkerk a/d Ijssel, The Netherlands) | Leiden University Medical Center (LUMC) and Amphia Hospital | Minimal gaps |
| Davies et al. 2018 | UK | H | NR | Retrospective | NR | Fresh samples were used and tested | NR | *C*. *difficile* toxin gene (tg) nucleic acid amplification tests (tgNAATs) were performed | PCR-ribotype 027 by the Cepheid Xpert *C. diff* assay | NR | Some gaps |
| De Francesco et al. 2019 | Italy | H/C | No | Retrospective | NR | Fresh samples were used and tested | NR | NR | Multiplex real-time PCR using Gene Xpert assay | Laboratory of Microbiology of Brescia-Spedali Civili Hospital | Many gaps |
| Dung et al. 2012 | Vietnam | H | Yes | At hospital admission | NR | All samples were stored at 4 ◦C before transportation to the laboratory on the same day as collection. Samples were stored at −80 ◦C. | Samples were stored as 10% suspensions in distilled phosphate-buffered saline (PBS). EAV was added to all samples prior to nucleic acid extraction as an internal control | Nucleic acid was extracted from bacterial and viral sources using the Wizard Nucleic Acid Purification Kit (Promega, USA) and the QIAamp Viral RNA Mini Kit (QIAGEN, USA), respectively | PCR amplifications were performed using RNA Master Hydrolysis Probes (Roche applied sciences, UK), and optimized with 1.4 μl of activator on a LightCycler 480II (Roche applied sciences,UK) | Microbiology laboratory of the Hospital for Tropical Diseases in HCMC | Minimal gaps |
| Elfving et al. 2014 | Zanzibar | C | Yes | Enrolment | Rectal swab samples were collected | Directly after rectal swab collection, the vials were placed in a vaccine carrier with a controlled temperature of 2 to 8°C. All swab samples were then stored at –70°C | Directly after sampling, the swabs were placed in sterile vials containing 1 ml of 0.9% NaCl within 2 hours after collection. 250 μL of the defrosted suspension was mixed with 2ml of lysis buffer | Nucleic acids were then extracted into 110 μL of elution buffer with a NucliSENS easyMAG robot (bioMérieux, Marcy l’Etoile, France) | Real-time PCR amplification was carried out in an ABI 7900 instrument (Applied Biosystems, Foster City, CA) | Diagnostic laboratory | No gaps |
| Feghaly et al. 2013a | USA | H | No | At hospital admission | NR | Stools were aliquoted and stored at −80◦C for later analysis | NR | Total nucleic acid was extracted using the NucliSENS  EasyMAG automated system software 1.0.2 specific A protocol (bioMérieux, Marcy L’Etoile, France). | Real-time PCR was performed using 7500 Fast Real-Time PCR System and Path-ID Multiplex One-Step RT-PCR mix (Applied Biosystems, Foster City, California) | Microbiology laboratory | Some gaps |
| Feghaly et al. 2013b | USA | H | Yes | Enrolment | NR | Stool samples were stored at −80◦C for later analysis | Stools were suspended in protease Inhibitor Lysis Buffer (Roche Diagnostics, Indianapolis, IN) and followed manufacturers' instructions for all EIAs. | Total nucleic acid was extracted using the NucliSENSR  EasyMAG™ automated system, software 1.0.2 specific A protocol (bioMerieux, Marcy L'Etoile, France), with a final volume of 110 μL of nucleic acid extract | Real-time reverse-transcription (RT)-PCR using Path-ID Multiplex one-step RT-PCR mix (Applied Biosystems, Foster City, CA) | Microbiology laboratory | Minimal gaps |
| Forsell et al. 2016 | Zanzibar | H | No | Enrolment | Faeces (150–200 mg) was collected with a flocked nylon swab (ESwab, Copan) | Two samples per patient were collected: one stored in L2-buffer containing guanidine thiocyanate and one stored in sodium acetate-acetic acid-formalin fixative (SAF). All fecal samples were stored at +4°C prior to parasite detection | Following collection, stool samples were transferred to 1 ml of Amies medium, 400 μL of this suspension was transferred to a tube with Lysing matrix E that contains beads (MP Biomedicals, Nordic Biolabs AB, Täby, Sweden) | DNA was extracted from samples stored in L2 buffer (guanidine-thiocyanate 0.96 g/ml dissolved in 0.1 M Tris, pH 6.4) using a method previously developed in the laboratory | *Entamoeba histolytica* and *Entamoeba dispar* were detected by a duplex qPCR assay. *Giardia intestinalis, Dientamoeba fragilis* and *Cryptosporidium spp*. were detected by a multiplex qPCR assay. | Clinical Microbiology laboratory, Umeå, Sweden | No gaps |
| Gustavsson et al. 2015 | Sweden | H | No | Retrospective | NR | NR | NR | NR | Real-time PCR | NR | NA, conference abstract |
| Haque et al. 2009 | Bangladesh | H | No | In hospital admission | NR | NR | Stool samples were cultured in Robinson’s medium within 6 h after collection. | For DNA extraction, DNA was purified from 200 mg of stool by use of the QIAamp DNA Stool Mini Kit (Qiagen). | *G. lamblia* genotypes were determined by use of a Scorpion probe–based real-time PCR assay. *Cryptosporidium* species were determined for stool specimens by use of a syber green-based real-time PCR technique that amplifies a *Cryptosporidium* coding sequence of unknown function (AF190627). All amplifications were performed according to references on a real-time PCR system (iCycler; BioRad) | NR | Some gaps |
| Hecht et al. 2009 | USA | H | Yes | Enrolment | NR | NR | Samples were aliquoted and frozen in Cary-Blair medium | NR | CD *tcdB* PCR assay | NR | NA, conference abstract |
| Jazmati et al. 2016 | Germany | H | NR | Retrospective | Loose stool sample | NR | NR | NR | Real-time PCR Cepheid GeneXpert) | NR | NA, letter/brief communication |
| Kabayiza et al. 2014a Pediatr Infect Dis | Rwanda | H/C | Yes | Enrolment | Rectal swab | Samples stored below +8°C for a few hours and were sent to a local laboratory for storage at −80°C until further testing | Samples were stored in a tube with 1 mL of sterile saline or as 2 mL of faeces | 250 μL of the dissolved feces or 250 μL of the rectal swab were mixed with 2 mL of lysis buffer, and this volume was used for extraction of  total nucleic acid in an EasyMag instrument (Biomerieux, Marcy  l’Étoile, France) | Real-time PCR was performed in an ABI7900 extraction of total nucleic acid in an EasyMag instrument (Biomerieux, Marcy l’Etoile, France) | Diagnostic laboratory | No gaps |
| Kabayiza et al. 2014b Clin Microbiol & Infec | Rwanda | H/C | NR | In hospital admission | Rectal swab | Samples were stored in a freezer at –80°C before molecular testing was performed | Samples added to a tube with 1 mL of sterile saline or as 2 mL of feces | Nucleic acid was extracted by the EasyMag instrument (Biomerieux, Marcy  l’Etoile, France) | Real-time PCR was performed in an ABI7900 extraction of total nucleic acid in an EasyMag instrument (Biomerieux, Marcy l’Etoile, France) | Diagnostic laboratory | No gaps |
| Kabue et al. 2016 | South Africa | C | Yes | Enrolment | By the clinic nurse | Stool specimens were kept at +4°C before transportation on ice to the laboratory within 6 h and stored at −20°C until tested | NR | The Boom method was used to extract NoV RNA. | - RIDA©GENE NOROVIRUS I & II real-time RT-PCR (r-BiopharmAG, Darmstadt,  Germany) kits were used to detect NoV from samples | University of Venda Microbiology laboratory | Minimal gaps |
| Kamboj et al. 2018 | USA | H | NR | Enrolment | All unformed stool samples were tested | Stool samples were frozen at −20°C until additional testing could be performed | NR | NR | Xpert *C. difficile* PCR | Blinded fashion | Some gaps |
| Kang et al. 2004 | India | H/C | Yes | For Rotavirus Detection EIA: in hospital admission, retrospective  Rotaviral Load Study Samples: enrolment | NR | NR | NR | Nucleic acid was extracted from 200 μL of 10% (100 μL of stool and 900 μL of phosphate buffered saline, pH 7.2) fecal suspensions using the guanidinium isothiocyanate-silica nucleic acid extraction method | cDNA was used in the VP6-specific PCR using the LightCycler™ (Roche, Mannheim, Germany) real-time PCR thermal cycler | NR | Many gaps |
| Liu et al. 2016 | Bangladesh, India, Pakistan, The Gambia, Kenya, Mali and Mozambique | C | Yes | Enrolment | NR | Stool samples were stored at –80°C. | NR | Nucleic acid was  extracted with the QIAamp Fast DNA Stool mini kit  (Qiagen, Hilden, Germany) with pretreatment steps that  included bead beating. | The performance of the molecular methods across three detection platforms were assessed: a PCR-Luminex strategy, multiplex real-time PCR panels, and a customised TaqMan array card. All laboratories used BioRad BioPlex 200 (BioRad, Hercules, CA) for detection except for Pakistan, which used MagPix (Luminex, Austin, TX) | Five laboratories in: Pakistan, The Gambia, Nepal, Tanzania and Bangladesh | Some gaps |
| Mukhopadhya et al. 2013 | India | H/C | Yes | In hospital admission/enrolment | By a trained field worker | NR | 10% of stool sample was suspended was diluted in balanced salt solution | Viral RNA was extracted from 200 μL the 10% fecal suspension using guanidium isothiocyanate and silica method | RT-qPCR using the SYBR green dye chemistry was performed using the real-time PCR Instrument Chromo4 system (MJ Research/Biorad, USA) | NR | Minimal gaps |
| Origuen et al. 2019 | Spain | H | No | In hospital admission/enrolment | NR | Unformed stools were stored in a container and processed immediately or, if that was logistically unfeasible, kept at 4°C for 24 to 48 h until processing | NR | NR | For samples with discordant results (GDH positive but toxin A/B negative), toxigenicity was confirmed by use of the Xpert *C. difficile* PCR assay (Cepheid, Sunnyvale, CA, USA), a real-time PCR assay targeting the *tcdB* gene of *C. difficile* | Microbiology laboratory; blinded fashion | Some gaps |
| Partridge et al. 2012 | UK | H | No | Retrospective | NR | NR | Faecal extracts are prepared using the Roche STAR Buffer | Nucleic acid was extracted using Roche MagNApure of Qiagen Qiacube systems | Amplification and detection of norovirus cDNA is carried out using the ABI prism 7,500 | Virology laboratory at located at NGH | Some gaps |
| Phillips et al. 2009 | UK | H | No | Enrolment | NR | Specimens with sufficient volume remaining after testing were  archived in frozen storage | NR | Norovirus RNA was re-extracted from the archived stored fecal specimens that were previously positive for norovirus by EM or RT-PCR, and real-time RT-PCR [additional references provided] | RT-PCR | Clinical virology laboratory | Many gaps |
| Phillips et al. 2009 | UK | H/C | No | Enrolment | NR | Specimens, with sufficient volume remaining after testing, were archived in frozen storage | NR | Norovirus RNA was re-extracted from the archived stored fecal specimens that were previously positive for norovirus by EM or RT-PCR, and real-time RT-PCR [additional references provided] | RT-PCR | NR | Many gaps |
| Ramani et al. 2010 | India | H | Yes | Enrolment | NR | NR | NR | Viral RNA was extracted from 10% fecal extracts from weighed stool samples using guanidine thiocyanate and silica | Real-time PCR was carried out using the SYBR green dye and primers VP6-F and VP6-R to amplify a 379-bp region of the VP6 gene [Iturriza Gomara et al., 2002] in the Chromo-4 real-time system (MJ Research/Bio-Rad, Waltham, MA) | NR | Many gaps |
| Rao et al. 2015 | USA | H | No | In hospital admission | NR | NR | Samples were sent to the laboratory in Cary-Blair transport medium, per hospital policy | NR | All GDH+/toxin– stool tests were subjected to analysis for the tcdB gene by real-time PCR using the GeneOhm Cdiff Assay (BD, Franklin Lakes, New Jersey) run on a Cepheid SmartCycler System (Cepheid, Sunnyvale, California) | Microbiology laboratory | Some gaps |
| Reigadas et al. 2016 | Spain | H | No | In hospital admission | By an investigator | For the derivation cohort, stool samples that yielded a CDI diagnosis were stored at –70°C until further testing | Stool samples transported  in formaldehyde or Cary–Blair medium | NR | Real-time PCR assay of the B toxin gene (Xpert^TM^ *C. difficile*) | NR | Minimal gaps |
| Saito et al. 2014 | Peru | C | Yes | At home visits | By a field worker | Samples were transported to UPCH within 12 hours for storage at −50°C | NR | RNA was extracted using silica particles with guanidinium thiocyanate | A segment of the ORF1–ORF2 junction was amplified by TaqMan real-time RT-PCR, using published primers and probes for genogroups I (GI) and II (GII) | Blinded fashion and not selected by genotype | Minimal gaps |
| Sante et al. 2018 | Spain | H | No | Enrolment | Samples were collected in a sterile bottle with no transport medium | Samples were stored at 2–8 ◦C until they were processed | Samples were collected in a sterile bottle with no transport medium | NR | Real-time PCR assay (Xpert^TM^ *C. difficile*) | Microbiology laboratory | NA, letter/brief communication |
| Vu et al. 2004 | Vietnam | C | No | Enrolment | Two rectal swab specimen by using a by using a cotton-tipped applicator (Solon Manufacturing Co., Solon, Maine) | The swab specimens were refrigerated until collection by a courier | One rectal swab specimen was inserted into Cary-Blair medium, and the other was inserted into buffered glycerol saline. The swabs were then transported in a cool box to the laboratory for testing | NR | The TaqMan real-time PCR assays were conducted with an ABI 7700 sequence detection system (Applied Biosystems) | Central laboratory at the Institute Pasteur, Nha Trang, Vietnam | Minimal gaps |

*C, community; H, hospital; NA, not applicable; NR, not reported; PCR, polymerase chain reaction; UK, United Kingdom; USA, United States of America.*
